# Degradation Characteristics of a Novel PAF Receptor Antagonist, SY0916, in Aqueous Solution

**DOI:** 10.1155/2019/8789470

**Published:** 2019-01-14

**Authors:** Bo Jin, Yanan Wang, Tingting Zhang, Wanting Yin, Dongfeng Zhang, Haihong Huang, Chen Ma

**Affiliations:** Institute of Material Medica, Chinese Academy of Medical Sciences and Peking Union Medical College, Beijing 100050, China

## Abstract

SY0916 has been proven to be a potent treatment agent against rheumatoid arthritis in preclinical studies and has been shown to be safe in phase I clinical trials. However, SY0916 is unstable in water, which is frequently used in pharmaceutical development processes. The degradation behaviour and stability of SY0916 in aqueous solutions were investigated at different pH levels, periods of time, and temperatures. Two degradation products (DPs) were successfully separated and characterized by liquid chromatography coupled to high-resolution tandem mass spectrometry (LC-HRMS/MS), liquid chromatography coupled to nuclear magnetic resonance with solid phase extraction (LC-SPE-NMR), and nuclear magnetic resonance (NMR). SY0916 decomposed to its *α*,*β*-unsaturated ketone in protonic solvents, and the *α*,*β*-unsaturated ketone further transformed into its alcohol form through a conjugate addition reaction in aqueous media. The results of this study indicate that the pH of the buffer solutions should be maintained between 3.0 and 3.6 for maximum SY0916 stability. Factors that affect degradation should be carefully controlled to mitigate or avoid drug decay.

## 1. Introduction

**Figure 1:**
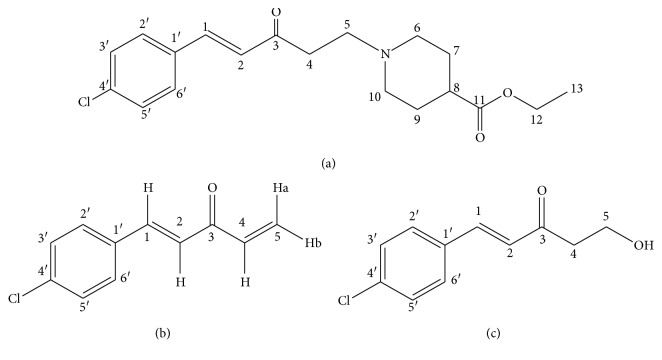
Structures of (a) SY0916, (b) D1, and (c) D2.

Platelet-activation factor (PAF) is considered a significant inflammatory mediator of different pathologies, including various types of inflammation [1, 2], allergy [3], and immune diseases [4, 5]. SY0916 ((E)-ethyl1-(5-(4-chlorophenyl)-3-oxopent-4-enyl)piperidine-4-carboxylate, Figure 1), a first-in-class PAF receptor antagonist, has been proven to be highly effective for rheumatoid arthritis therapy in preclinical studies and has been shown to be safe in phase I clinical trials [6–11]. Unlike commercially available antiarthritis drugs, which have the side effect of causing gastrointestinal injury, SY0916 possesses the notable advantage of protecting gastric mucosa [6]. Moreover, in rats with collagen-induced type II arthritis, SY0916 significantly relieved inflammation in soft tissue around the middle of joints, as observed by X-ray and pathological examination [7], and greatly decreased the serum levels of IgG, TNF-*α*, and IL-*β* [8]. In a pharmacokinetic study in healthy humans [7], SY0916 was absorbed rapidly (t_1/2_ = 0.5 h ± 0.1 h) and was quickly eliminated in its metabolite form through renal excretion. Furthermore, doses of up to 500 mg were well tolerated and presented no serious adverse events in the first-in-human study.

SY0916 is slightly soluble in water but easily decomposes into two unknown degradation products (DPs) in water. Because water and other aqueous solutions are frequently introduced during various pharmaceutical development processes, the underlying degradation mechanism of SY0916 in aqueous solutions must be elucidated. The aim of this study was to identify the DPs and clarify the degradation characteristics of SY0916 in water and other aqueous solutions. Accordingly, structural characterization of the DPs was performed using liquid chromatography coupled to high-resolution tandem mass spectrometry (LC-HRMS/MS), liquid chromatography coupled to nuclear magnetic resonance with solid phase extraction (LC-SPE-NMR), and nuclear magnetic resonance (NMR). The stability of SY0916 in solution was controlled by using a specific pH range and time. Based on the structural identification and stability studies of the DPs, the degradation mechanism of SY0916 in aqueous solutions was elucidated, and relevant measures aimed at avoiding drug decay were proposed.

## 2. Materials and Methods

### 2.1. Materials and Chemicals

Bulk drug substance SY0916 was provided by the Organic Synthesis Lab at the Institute of Materia Medica, Chinese Academy of Medical Sciences & Peking Union Medical College (Beijing, China). (4E)-5-(4-chlorophenyl) penta-1,4-dien-3-one (D1; 95%) was synthesized by Chemspace SIA (Riga, Latvia). HPLC-grade acetonitrile, phosphoric acid (85%), and ammonium acetate were purchased from Fisher Scientific Products (Fair Lawn, NJ, USA). HPLC-grade acetic acid was obtained from Tedia Company, Inc (Fairfield, OH, USA). Analytical-grade monopotassium phosphate was purchased from Beijing Chemical Works (Beijing, China). Methanol-d4 (CD_3_OD, 99.95%) was obtained from Sigma-Aldrich Co. (St. Louis, MO, USA). Ultrapure water was obtained from a Milli-Q water purification system (Millipore, Bedford, MA, USA).

### 2.2. HPLC Analysis

The separation of SY0916 and its DPs was achieved using a Shimadzu LC-20AD liquid chromatography system with an autoinjector, binary solvent manager, and photodiode array (PDA) detector (Shimadzu, Tokyo, Japan). The column was an Alltima Cyano 100A column (4.6 × 250 mm, 5 *µ*m, Thermo Scientific, MA, USA), and the oven temperature was set to 30°C. The mobile phase was a mixture of a 0.006 mol/L ammonium acetate solution with 0.22% acetic acid and acetonitrile (pH 3.9, 70 : 30, v/v). The flow rate was set to 1.0 mL/min, the injection volume was 20 *μ*L, and the detector wavelength was 300 nm.

### 2.3. LC-HRMS/MS Analysis

All LC-HRMS/MS data were collected using an Agilent 1290 Infinity UHPLC coupled to a 6540 UHD Accurate-Mass Q-TOF mass spectrometer with an electrospray ionization source (Agilent Technologies, CA, USA) operated in a positive ion mode. MassHunter Qualitative Analysis B.06.00 was used to control the system and to perform data acquisition and processing. HPLC was performed as described above, with the flow rate decreased to 0.5 mL/min. The optimized source conditions for the MS scans in positive ion mode (ESI+) were as follows: an ion spray voltage of 3500 V, nozzle voltage of 1000 V, carrier gas temperature of 300°C, carrier gas flow of 8 L/min, sheath gas temperature of 350°C, sheath gas flow of 11 L/min, fragmentor voltage of 135 V, and collision energy of 20 eV for D1 and 15 eV for D2. Due to its high concentration as well as the possibility of it contaminating the mass analyser, the HPLC fraction corresponding to SY0916 was diverted to waste by switching the instrument valve at a set time.

### 2.4. NMR Analysis of D1

One- and two-dimensional NMR spectra (^1^H NMR, ^13^C NMR, heteronuclear single quantum coherence (HSQC), and heteronuclear multiple bond correlation (HMBC)) of synthesized D1 were recorded on a Bruker AVANCE III HD 600 MHz spectrometer (Bruker BioSpin, Rheinstetten, Germany). The sample was dissolved in CD_3_OD, and tetramethylsilane was used as the chemical shift reference standard.

### 2.5. LC-SPE-NMR Analysis of D2

Twenty millilitres of a 1 mg/mL SY0916 solution in monopotassium phosphate buffer (pH 5.0) was heated in a water bath at 80°C for 24 h to obtain a stressed solution of D2. The stressed solution was extracted three times with 5 mL of ethyl acetate each time, dried under nitrogen at room temperature, and dissolved in 4 mL of acetonitrile; the resulting solution was used for LC-SPE-NMR analysis.

One- and two-dimensional NMR spectra (^1^H NMR, ^13^C NMR, HSQC, and HMBC) of D2 were obtained using the LC-SPE-NMR system. LC-SPE-NMR analysis was performed on a Bruker AVANCE III HD 600 MHz spectrometer (Bruker BioSpin, Rheinstetten, Germany) coupled with an Agilent 1260 system (Agilent Technology, CA, USA). The resulting solution was separated using the HPLC method described in Section 2.2. Based on its ultraviolet (UV) spectrum and peak retention time, the component of interest was collected by a HySphere Resin GP10 cartridge after the postcolumn addition of water using a Knauer K100 HPLC pump (Berlin, Germany). The trapped analyte was dried under N_2_ gas for 55 min and eluted at 0.2 mL/min with CD_3_OD into a 3-mm NMR tube for NMR analysis. The chemical shifts were recorded in ppm (*δ*) downfield from the internal standard, tetramethylsilane (TMS). Bruker TopSpin software version 3.2 was used for data acquisition and processing.

### 2.6. Degradation and Stability Studies

Sample solutions were prepared by dissolving SY0916 in water (pH 6.2) and phosphate buffers at pH 3.0, pH 3.2, pH 3.5, pH 3.6, pH 4.0, and pH 5.0 to obtain a final concentration of 1 mg/mL. The pH was measured using an FE28 pH metre (Mettler-Toledo, Switzerland). The solutions were kept at room temperature and 10°C ± 1°C, with the temperature maintained by the built-in temperature-controllable specimen chamber of the HPLC instrument. Working samples were withdrawn at 0 h, 2 h, 4 h, 6 h, 8 h, 10 h, 12 h, 14 h, 16 h, 18 h, and 24 h and filtered through a 0.45 *µ*m membrane syringe filter (ANPEL Laboratory Technologies Inc., Shanghai, China).

## 3. Results and Discussion

### 3.1. Degradation and Stability Studies

**Figure 2:**
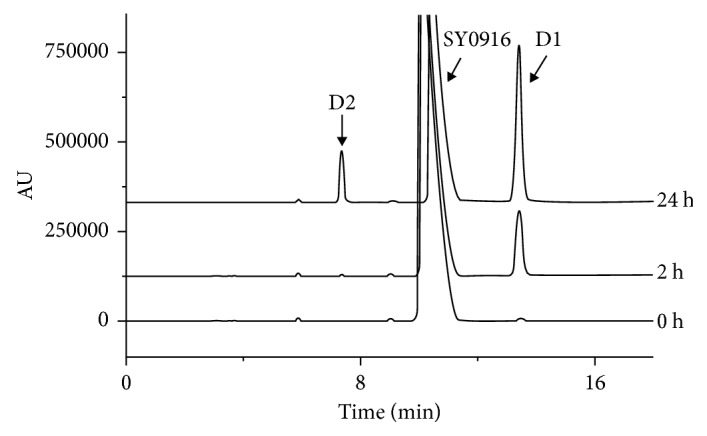
HPLC chromatographs of sample solutions at 0 h, 2 h, and 24 h in water at room temperature.

SY0916 is slightly soluble in water according to the USP classification of solutes. A bulk sample of SY0916 was dissolved in water. The solution was kept at room temperature for 24 h and was assessed by HPLC in discrete intervals. Two unknown DPs (D1 and D2) were observed (Figure 2). SY0916 degradation was apparent at as early as 2h in water, at which time the major DP was D1 (7.6%). At 24 h, 23.2% of the DPs was D1, and 4.1% was D2.

The stability of SY0916 in buffer solutions at pH 3.0∼5.0 was further studied using HPLC in discrete intervals. At room temperature, SY0916 was stable for 4h in buffer solutions at pH 3.0∼3.6 (99.76% ± 0.05%, calculated by a normalization method). At low temperature (10°C ± 1°C), samples in solutions at pH 3.0∼3.6 remained stable for a longer time period (24 h) than did the samples at room temperature.

**Figure 3:**
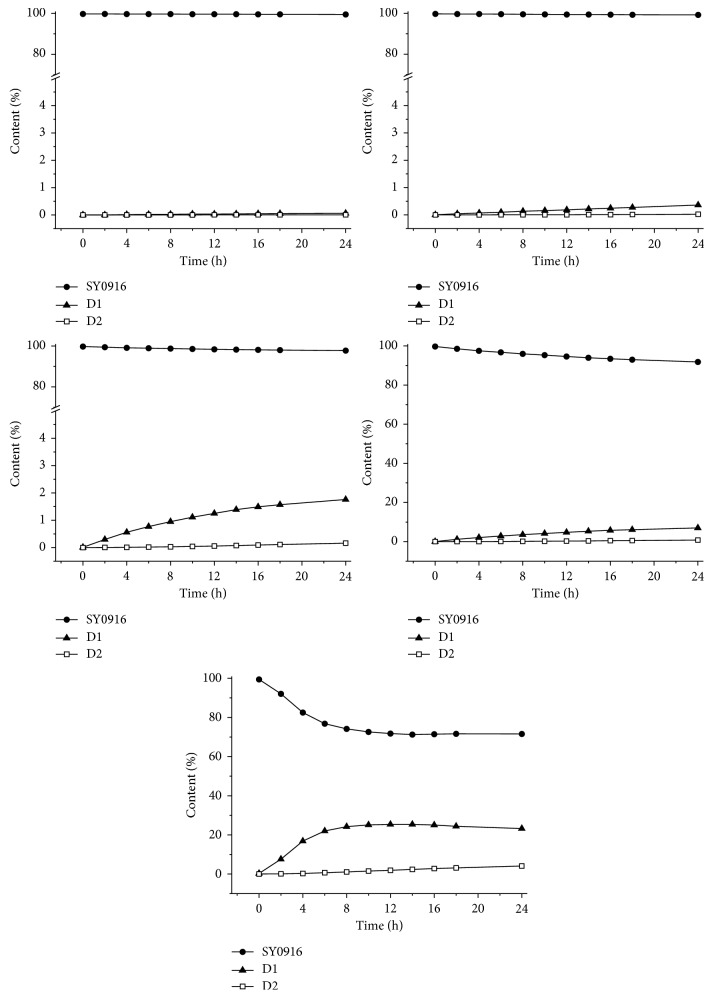
Content of SY0916, D1, and D2 in buffer solutions (pH 3.0∼5.0) and water over 24 h at room temperature.

As shown in Figure 3, more D1 was produced in the aqueous solutions with higher pH levels (pH ≥ 4.0 and water). Compared with D1 in the same sample solutions, the D2 formation rate was much slower, and the yield was lower. Similarly, aqueous solutions at higher pH favoured the production of D2.

### 3.2. Structure Elucidation of the Degradation Products

#### 3.2.1. D1

**Figure 4:**
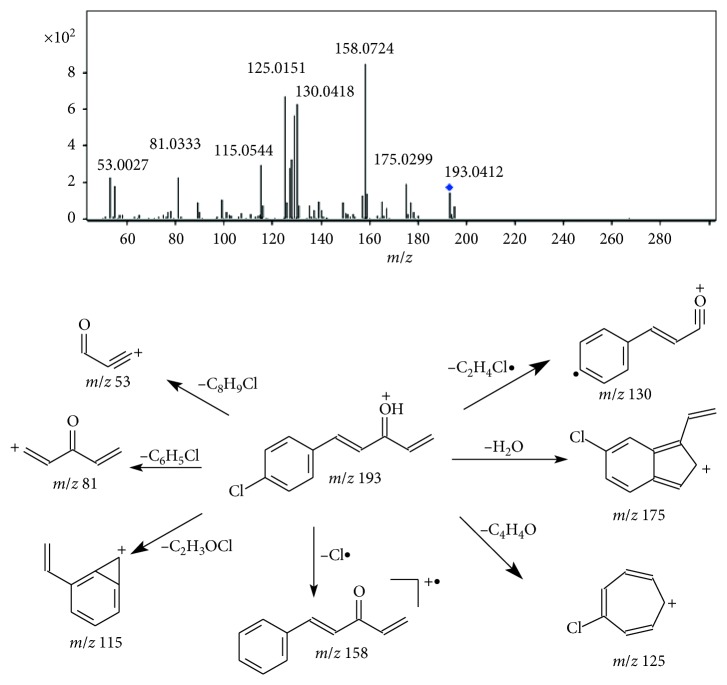
LC-HRMS/MS spectrum and plausible fragmentation pathway of protonated D1.

*(1) LC-HRMS/MS Analysis of D1*. The LC-HRMS/MS spectrum of D1 exhibited a protonated molecular ion at *m*/*z* 193, which is by *m*/*z* 157 mass units lower than protonated SY0916 and possibly results from the loss of the 4-carboxylate piperidine moiety. As shown in Figure 4 and Table 1, the MS^2^ spectrum of the precursor ion at *m*/*z* 193 displayed a prominent product ion at *m*/*z* 158 due to the loss of the Cl radical (35 Da). The product ion at *m*/*z* 175 was proposed to be the product after the loss of H_2_O (18 Da) from *m*/*z* 193 [12]. The product ion at *m*/*z* 130 was assumed to be formed after the loss of the Cl radical (35 Da) and a molecule of ethylene (28 Da) from the precursor ion. The product ion at *m*/*z* 125 was considered a chlorine-substituted tropylium ion due to the loss of C_4_H_4_O (58 Da) from the ion at *m*/*z* 193. The ion at *m*/*z* 115 was speculated to be an ylium, as illustrated in Figure 4 [13, 14]. Moreover, the fragment ion at *m*/*z* 81 resulted from the loss of the 4-chlorophenyl moiety from the precursor ion. The ion at *m*/*z* 53 was due to the loss of one molecule of 1-chloro-4-ethylbenzene (140 Da) from the precursor ion. Therefore, we tentatively deduced that the chemical structure of D1 is (4E)-5-(4-chlorophenyl) penta-1,4-dien-3-one (Figure 1). The proposed fragmentation pathway is shown in Figure 4. Synthesized D1 was validated by MS analysis under the conditions described in Section 2.3, and the acquired data were in agreement with those of D1 in the sample solutions.

*(2) NMR Analysis of D1*. The atom assignments for the ^1^H and ^13^C chemical shifts of D1 are summarized in Table 2. ^1^H protons were assigned to three separate spin systems. The *δ*_H_ 7.62∼7.60 (m) and *δ*_H_ 7.37∼7.36 (m) of H-2′ (H-6′) and H-3′ (H-5′) indicated the presence of a parasubstituted phenyl subunit. The spin system incorporated protons at 7.64 ppm (H-1) and 7.11 ppm (H-2) with a coupling constant of 16.1 Hz, indicating the presence of a trans-vinyl subunit. The peaks at 6.73 ppm (H-4), 6.37 ppm (H-5a), and 5.89 ppm (H-5b) with three pairwise coupling constants (17.5 Hz, 10.7 Hz, 1.2 Hz) showed a typical terminal vinyl subunit. The HSQC correlation peaks of synthesized D1 provided decisive chemical shift assignments of the carbons directly coupled to protons, which was especially helpful for some carbons with similar chemical shifts, such as C-2′ (C-6′) and C-3′ (C-5′). HMBC correlated H-3′ (H-5′) and H-2 with C-1′ at 134.85 ppm and the H-2′ (H-6′) with C-4′ at 137.54 ppm, which offered conclusive evidence for the atom assignments of C-1′ and C-4′.

#### 3.2.2. D2

**Figure 5:**
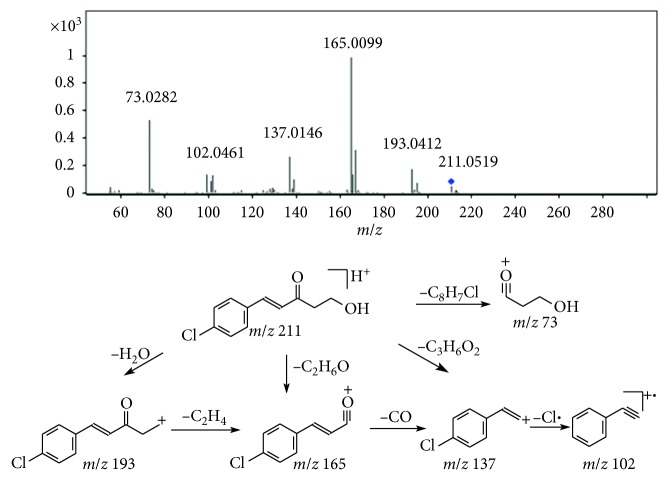
LC-HRMS/MS spectrum and plausible fragmentation pathway of protonated D2.

*(1) LC-HRMS/MS Analysis of D2*. As shown in Figure 5 and Table 1, the protonated molecular ion [M + H]^+^ was observed at *m*/*z* 211 and exhibited a mass that better corresponded to H_2_O than to D1, suggesting that one of the ketene double bonds of D1 was opened to add a H_2_O molecule and form D2. The product ion at *m*/*z* 193 was proposed to be the product after the loss of H_2_O (18 Da) from the *m*/*z* 211 ion. The MS^2^ spectrum of the precursor ion featured major product ions at *m*/*z* 165, 137, and 102, indicating that the addition reaction occurred at the terminal double bonds, which successively lost C_2_H_6_O (46 Da), CO (28 Da), and the Cl radical (35 Da). This conclusion was confirmed by an MS^3^ experiment (Supplementary Figure A). The product ion at *m*/*z* 73 was the complementary part of the product ion at *m*/*z* 137 from the precursor ion at *m*/*z* 211. The proposed structure of D2 is (E)-1-(4-chlorophenyl)-5-hydroxypent-1-en-3-one (Figure 1), and the fragmentation pathway of D2 is shown in Figure 5.

*(2) LC-SPE-NMR analysis of D2*. The atom assignments for the ^1^H and ^13^C chemical shifts are listed in Table 2. Similar to D1, the protons of D2 were assigned to three separate spin systems. The appearance of protons at 7.37 ppm and 7.58∼7.61 ppm with a coupling constant of 8.5 Hz indicated the presence of a para-substituted phenyl subunit. The second spin system involved protons at 7.55 ppm and 6.81 ppm with a coupling constant of 16.3 Hz, indicating the presence of a trans-vinyl subunit. The peaks at 3.84 ppm and 2.87 ppm showed three splits and a coupling constant of 6.1 Hz, suggesting the presence of an ethanol subunit. In addition, the HSQC correlation peaks provided decisive chemical shift assignments of the carbons with similar chemical shifts, such as C-2′ (C-6′) and C-3′ (C-5′). HMBC correlated H-3′ (H-5′) with C-1′ at 134.79 ppm and correlated H-2′ (H-6′) with C-4′ at 137.41 ppm, which offered conclusive evidence for the atom assignments of C-1′ and C-4′. The LC-SPE-NMR results confirmed the structure of D2 that was proposed by LC-HRMS/MS.

### 3.3. Degradation Mechanisms of SY0916 in Aqueous Solutions

Based on the unambiguous structures of the degradants, the degradation pathway of SY0916 in solution was proposed. SY0916 is a Mannich base that tends to decompose to its *α,β*-unsaturated ketone (D1) under alkaline conditions. Furthermore, the *α*,*β*-unsaturated ketone may not be stable in aqueous solutions and is likely to transform to its alcohol form (D2) through a conjugate addition reaction.

With a better understanding of the formation mechanisms of the degradants, special attention should also be paid to the primary factors affecting degradant levels. Water, solutions with high pH, and protonic solvents such as alcohols should be used with care to prevent the degradation of SY0916 to D1. A large quantity of aqueous solution, especially at high temperatures, should be avoided to prevent the introduction of D2. The stability of SY0916 in aqueous solution depends on the pH of the solution. As described above, the results of this study provide a useful reminder to consider the conditions during SY0916 development processes, such as the dissolvent choice for analytical methods in routine assays or for pharmacokinetic studies and the solvent use in formula preparations.

## 4. Conclusions

With the help of LC-HRMS/MS, LC-SPE-NMR, and NMR approaches, structures of the two degradants of SY0916 were rapidly characterized, and their degradation mechanism in sample solutions was elucidated. The drug substance decomposed to its *α*,*β*-unsaturated ketone in protonic solvents, and the *α*,*β*-unsaturated ketone further transformed to its alcohol form through a conjugate addition reaction in aqueous media. Systematic stability studies of sample solutions showed that the pH of buffer solutions should be between 3.0 and 3.6 for maximum SY0916 stability. Factors affecting the degradant levels should be carefully controlled during the procedures used in various pharmaceutical development processes.

## Figures and Tables

**Table 1:** Elemental composition of the D1 and D2 product ions.

Degradation products	Measured mass (*m*/*z*)	Calculated mass (*m*/*z*)	Proposed molecular formula	Error (ppm)	Proposed loss
D1	193.0412	193.0415	C_11_H_10_ClO^+^	−1.55	
	175.0299	175.0309	C_11_H_8_Cl^+^	−5.71	-H_2_O
	158.0724	158.0726	C_11_H_10_O^+˙^	−1.27	-Cl^˙^
	130.0418	130.0413	C_9_H_6_O^+˙^	3.84	-C_2_H_4_Cl^˙^
	125.0151	125.0152	C_7_H_6_Cl^+^	−0.80	-C_4_H_4_O
	115.0544	115.0542	C_9_H_7_^+^	1.74	-C_2_H_3_OCl
	81.0333	81.0334	C_5_H_5_O^+^	−1.23	-C_6_H_5_Cl
	53.0027	53.0022	C_3_HO^+^	9.43	-C_8_H_9_Cl

D2	211.0519	211.0520	C_11_H_12_O_2_Cl^+^	−0.47	
	193.0412	193.0414	C_11_H_10_OCl^+^	−1.04	-H_2_O
	165.0099	165.0101	C_9_H_6_OCl^+^	−1.21	-H_2_O-C_2_H_4_
	137.0146	137.0152	C_8_H_6_Cl^+^	−4.38	-H_2_O-C_2_H_4_-CO
	102.0461	102.0464	C_8_H_6_^+˙^	−2.94	-H_2_O-C_2_H_4_-CO-Cl^˙^
	73.0282	73.0284	C_3_H_5_O_2_^+^	−2.74	-C_8_H_7_Cl

**Table 2:** ^1^H and ^13^C chemical shifts (ppm) and ^1^H coupling data of D1 from NMR and of D2 from LC-SPE-NMR.

Position	D1				D2			
	*δ*H (ppm)	*δ*C (ppm)	HSQC	HMBC	*δ*H (ppm)	*δ*C (ppm)	HSQC	HMBC
1	7.64 (d, *J* = 16.1 Hz)	144.03	H-1	H-2′(6′), H-2	7.55 (d, *J* = 16.3 Hz)	143.12	H-1	H-2′(6′)
2	7.11 (d, *J* = 16.1 Hz)	125.71	H-2	H-1	6.81 (d, *J* = 16.3 Hz)	128.07	H-2	—
3	—	191.47	—	H-1, H-2, H-4, H-5 (Ha), H-5 (Hb)	—	201.20	—	H-1, H-2, H-4, H-5
4	6.73 (dd, *J* = 17.5 Hz, 10.7 Hz)	136.58	H-4	H-5 (Ha)	2.87 (t, *J* = 6.1 Hz)	44.10	H-4	H-5
5	6.37 (Ha, dd, *J* = 17.5 Hz, 1.2 Hz)	129.86	H-5 (Ha)	—	3.84 (t, *J* = 6.1 Hz)	58.50	H-5	H-4
	5.89 (Hb, dd, *J* = 10.7 Hz, 1.2 Hz)		H-5 (Hb)					
1′	—	134.85	—	H-2, H-3′(5′)	—	134.79	—	H-3′(5′)
2′	7.62–7.60 (m)	131.05	H-2′ (H-6′)	H-1	7.61-7.58 (m)	130.93	H-2′ (H-6′)	H-1, H-3′(5′)
3′	7.37-7.36 (m)	130.24	H-3′ (H-5′)	—	7.37 (d, *J* = 8.5 Hz)	130.23	H-3′ (H-5′)	H-2′(6′)
4′	—	137.54	—	H-2′(6′), H-3′(5′)	-	137.41	-	H-2′(6′)
5′	7.37-7.36 (m)	130.24	H-5′ (H-3′)	—	7.37 (d, *J* = 8.5 Hz)	130.23	H-5′ (H-3′)	H-2′(6′)
6′	7.62–7.60 (m)	131.05	H-6′ (H-2′)	H-1	7.61-7.58 (m)	130.93	H-6′ (H-2′)	H-1, H-3′(5′)

## Data Availability

All data included in this study are available upon request from the corresponding author.

## Supplementary Materials

Supplementary Materials (1) The supplementary data Figure A includes Figures A.1–3 as follows: Figure A.1 (MS^3^ spectrum of *m*/*z* 193 ion from *m*/*z* 211 of D2), Figure A.2 (MS^3^ spectrum of *m*/*z* 165 ion from *m/z* 211 of D2), and Figure A.3 (MS^3^ spectrum of *m*/*z* 137 ion from *m*/*z* 211 of D2). Figures A. 1–3 were the supporting spectra to LC‐HRMS/MS analysis of D2 in Section 3.2.2 of the submitted manuscript: “The product ion at *m*/*z* 193 was proposed to be the product after the loss of H_2_O (18 Da) from *m*/*z* 211. The MS^2^ spectrum of the precursor ion featured major product ions at *m*/*z* 165, 137, and 102, indicating the addition reaction position was on the terminal double bonds, which successively lose C_2_H_6_O (46 Da), CO (28 Da), and Cl radical (35 Da). It was confirmed by a MS_3_ experiment (supplementary data Figure A).” The supplementary data Figure A was annotated in the submitted manuscript. (2) The supplementary data Figure B and Figure C correspond to the NMR data listed in Table 2. The supplementary data was not annotated in Table 2 of the submitted manuscript. Figure B includes six figures as follows: Figure B.1 1H spectrum of synthesized D1; Figure B.2 13C spectrum of synthesized D1; Figure B.3 HSQC spectrum of synthesized D1; Figure B.4 partial-enlarged HSQC spectrum of synthesized D1; Figure B.5 HMBC spectrum of synthesized D1; and Figure B.6 partial-enlarged HMBC spectrum of synthesized D1. Figures B.1–6 were original spectra of the 1H and 13C chemical shifts and HSQC and HMBC information of D1. Figure C includes six figures as follows: Figure C.1 1H spectrum of D2 by LC-SPE-NMR; Figure C.2 13C spectrum of D2 by LC-SPE-NMR; Figure C.3 HSQC spectrum of D2 by LC-SPE-NMR; Figure C.4 partial-enlarged HSQC spectrum of D2 by LC-SPE-NMR; Figure C.5 HMBC spectrum of D2 by LC-SPE-NMR; and Figure C.6 partial-enlarged HMBC spectrum of D2 by LC-SPE-NMR. Figures C.1–6 were original spectra of the 1H and 13C chemical shifts and HSQC and HMBC information of D2.
