# Supplementary material for: Degradation Characteristics of a Novel PAF Receptor Antagonist, SY0916, in Aqueous Solution
Source: J Anal Methods Chem. 2019 Jan 14;2019:8789470. doi: 10.1155/2019/8789470 (PMC6350595; doi:10.1155/2019/8789470)
Supplement: Supplementary Materials — (1) The supplementary data Figure A includes Figures A.1–3 as follows: Figure A.1 (MS3 spectrum of m/z 193 ion from m/z 211 of D2), Figure A.2 (MS3 spectrum of m/z 165 ion from m/z 211 of D2), and Figure A.3 (MS3 spectrum of m/z 137 ion from m/z 211 of D2). Figures A. 1–3 were the supporting spectra to LC‐HRMS/MS analysis of D2 in Section 3.2.2 of the submitted manuscript: “The product ion at m/z 193 was proposed to be the product after the loss of H2O (18 Da) from m/z 211. The MS2 spectrum of the precursor ion featured major product ions at m/z 165, 137, and 102, indicating the addition reaction position was on the terminal double bonds, which successively lose C2H6O (46 Da), CO (28 Da), and Cl radical (35 Da). It was confirmed by a MS3 experiment (supplementary data Figure A).” The supplementary data Figure A was annotated in the submitted manuscript. (2) The supplementary data Figure B and Figure C correspond to the NMR data listed in Table 2. The supplementary data was not annotated in Table 2 of the submitted manuscript. Figure B includes six figures as follows: Figure B.1 1H spectrum of synthesized D1; Figure B.2 13C spectrum of synthesized D1; Figure B.3 HSQC spectrum of synthesized D1; Figure B.4 partial-enlarged HSQC spectrum of synthesized D1; Figure B.5 HMBC spectrum of synthesized D1; and Figure B.6 partial-enlarged HMBC spectrum of synthesized D1. Figures B.1–6 were original spectra of the 1H and 13C chemical shifts and HSQC and HMBC information of D1. Figure C includes six figures as follows: Figure C.1 1H spectrum of D2 by LC-SPE-NMR; Figure C.2 13C spectrum of D2 by LC-SPE-NMR; Figure C.3 HSQC spectrum of D2 by LC-SPE-NMR; Figure C.4 partial-enlarged HSQC spectrum of D2 by LC-SPE-NMR; Figure C.5 HMBC spectrum of D2 by LC-SPE-NMR; and Figure C.6 partial-enlarged HMBC spectrum of D2 by LC-SPE-NMR. Figures C.1–6 were original spectra of the 1H and 13C chemical shifts and HSQC and HMBC information of D2. [file 8789470.f1.pdf]

Degradation characteristics of a novel PAF receptor antagonist SY0916 in aqueous solution

Supplementary data

Fig. A The MS^3 spectra of D2

Fig.A.1 MS^3 spectrum of m/z 193 ion from m/z 211 of D2

Fig.A.2 MS^3 spectrum of m/z 165 ion from m/z 211 of D2

Fig.A.3 MS^3 spectrum of m/z 137 ion from m/z 211 of D2

Fig. B The NMR spectra of D1

Fig.B.1 ^1H spectrum of synthesized D1

Fig.B.2 ^{13}C spectrum of synthesized D1

Fig.B.3 HSQC spectrum of synthesized D1

Fig.B.4 partial-enlarged HSQC spectrum of synthesized D1

Fig.B.5 HMBC spectrum of synthesized D1

Fig.B.6 partial-enlarged HMBC spectrum of synthesized D1

Fig. C The NMR spectra of D2

Fig.C.1 ^1H spectrum of D2 by LC-SPE-NMR

Fig.C.2 ^{13}C spectrum of D2 by LC-SPE-NMR

Fig.C.3 HSQC spectrum of D2 by LC-SPE-NMR

Fig.C.4 partial-enlarged HSQC spectrum of D2 by LC-SPE-NMR

Fig.C.5 HMBC spectrum of D2 by LC-SPE-NMR

Fig.C.6 partial-enlarged HMBC spectrum of D2 by LC-SPE-NMR

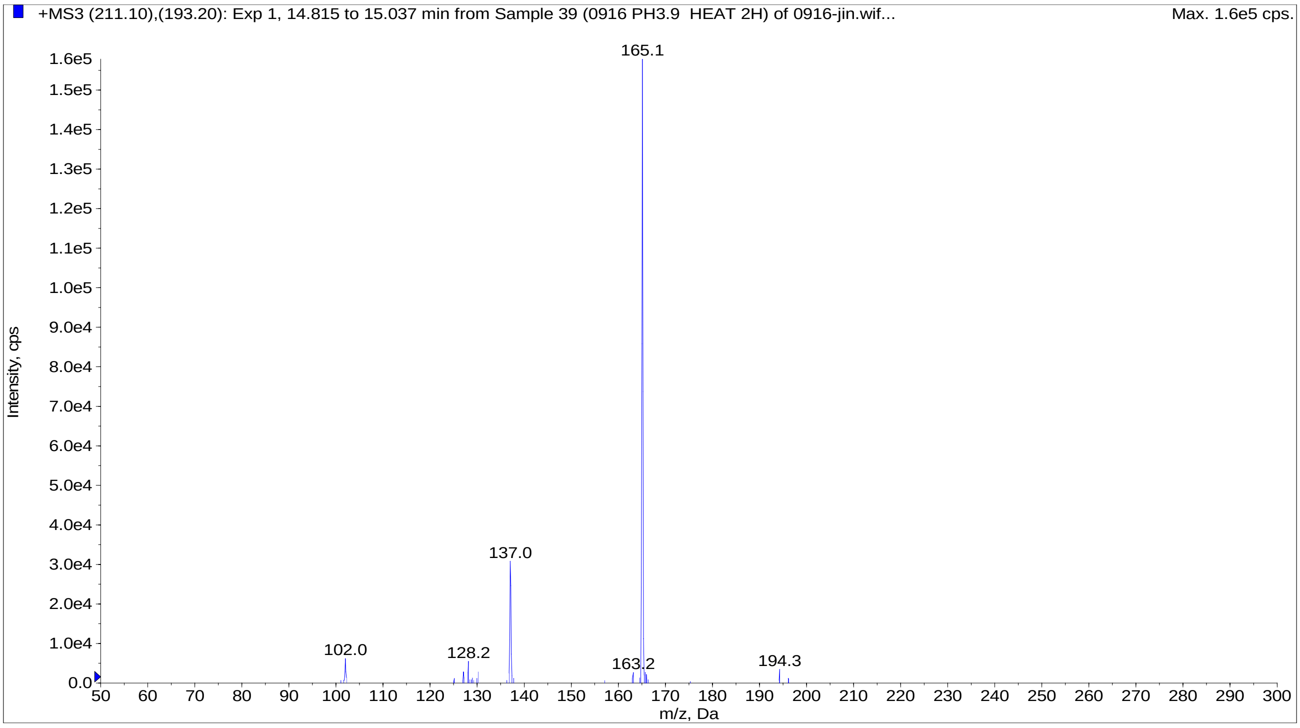

Fig.A.1 MS^3 spectrum of m/z 193 ion from m/z 211 of D2

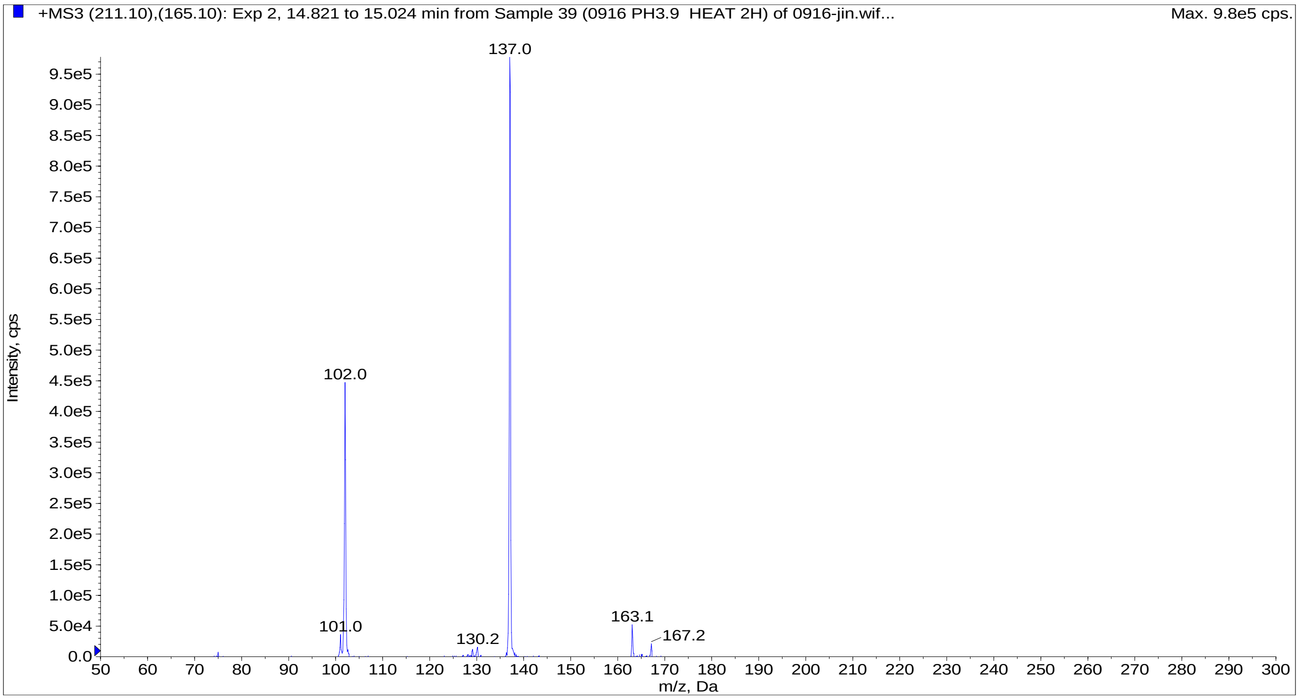

Fig.A.2 MS^3 spectrum of m/z 165 ion from m/z 211 of D2

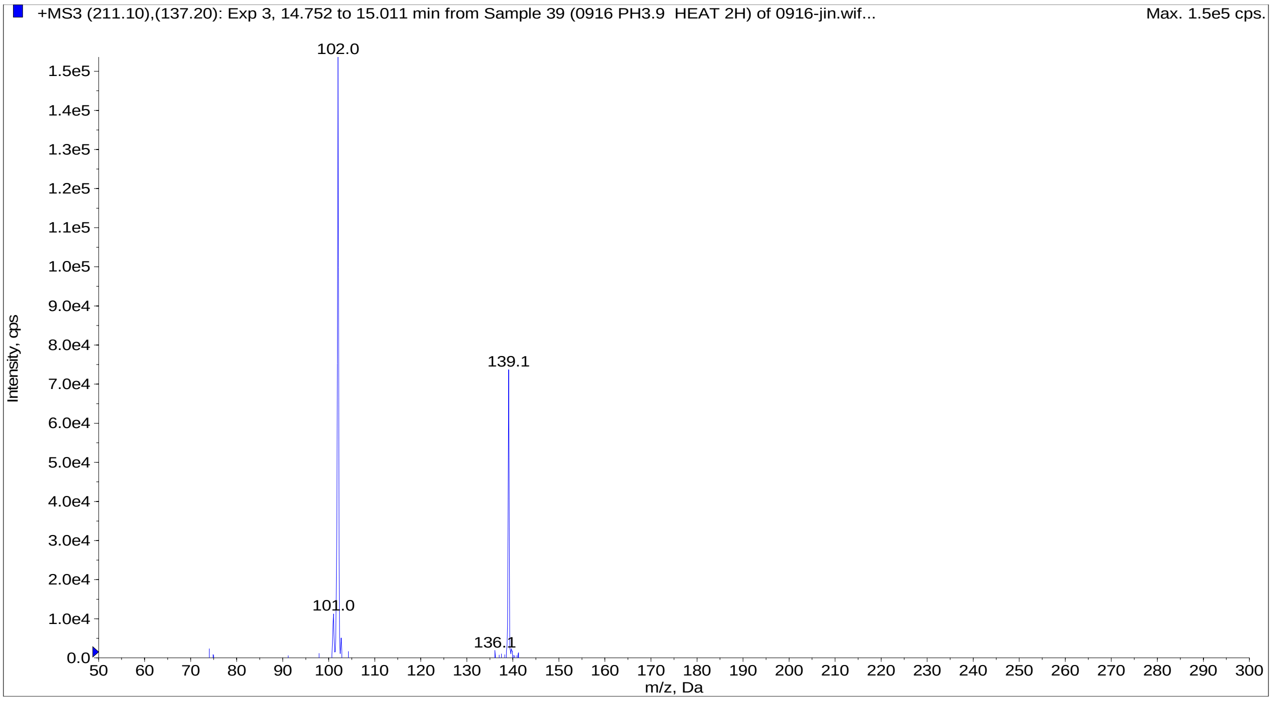

Fig.A.3 MS^3 spectrum of m/z 137 ion from m/z 211 of D2

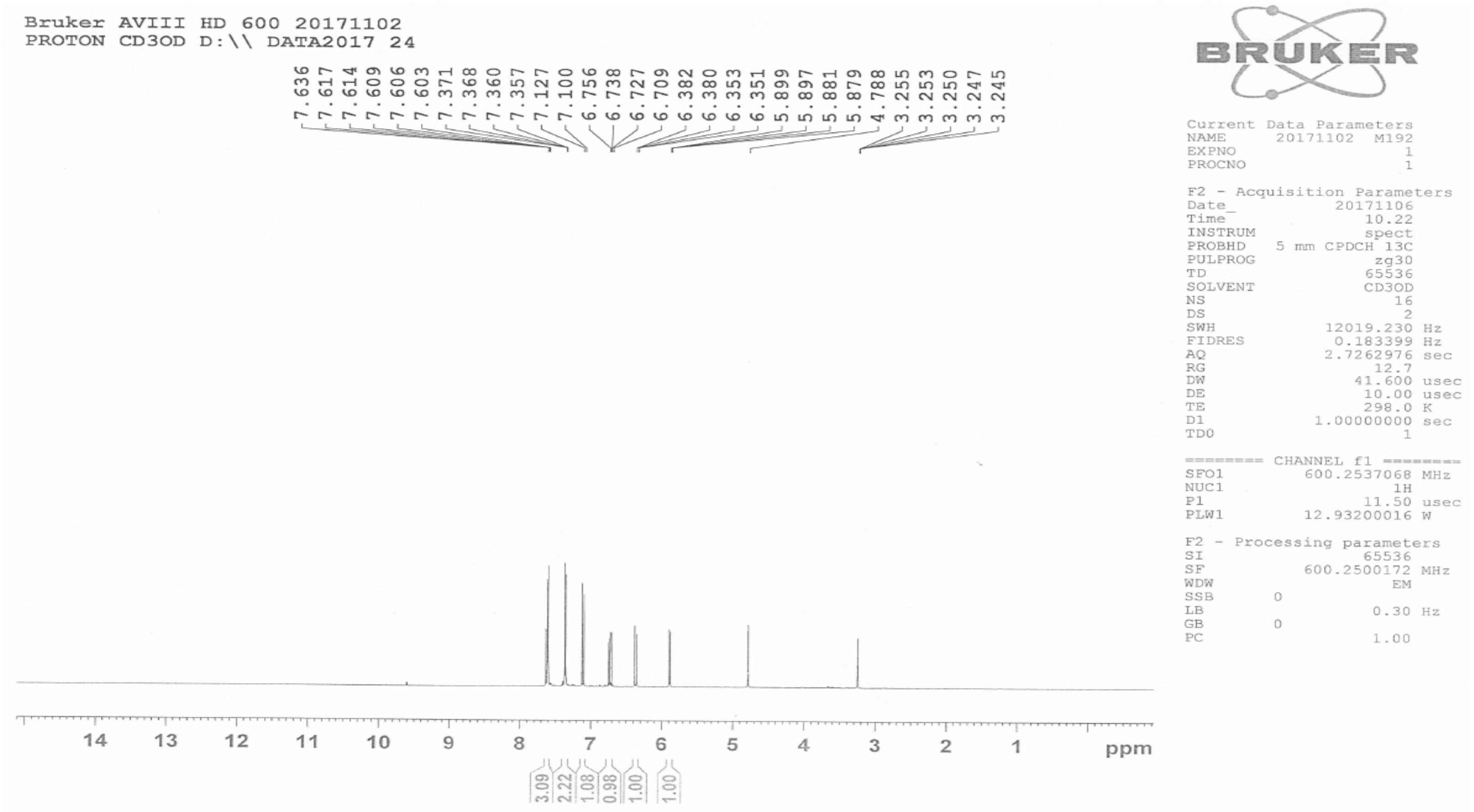

Fig.B.1 ^{1}H spectrum of synthesized D1

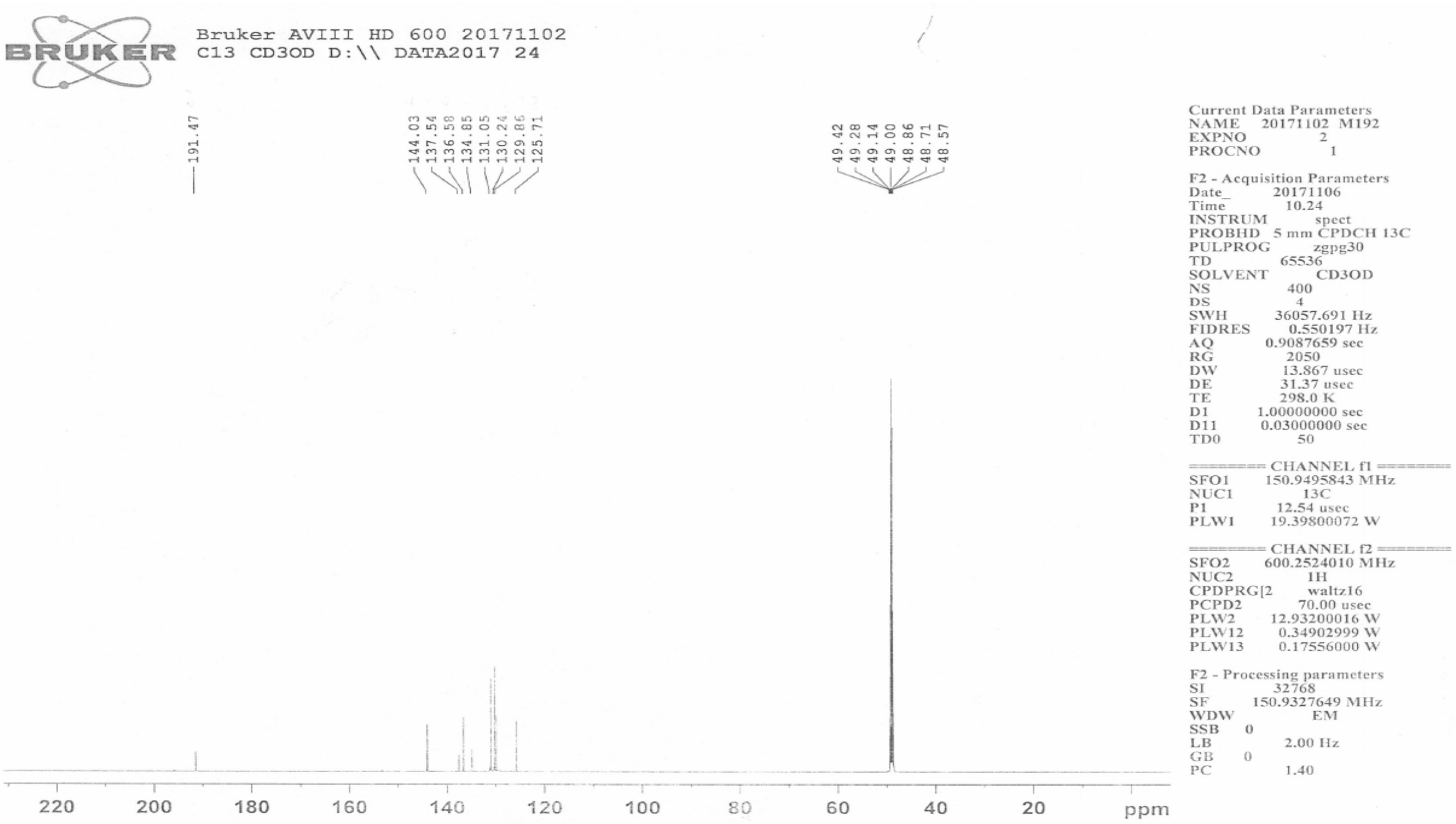

Fig.B.2 ^{13}C spectrum of synthesized D1

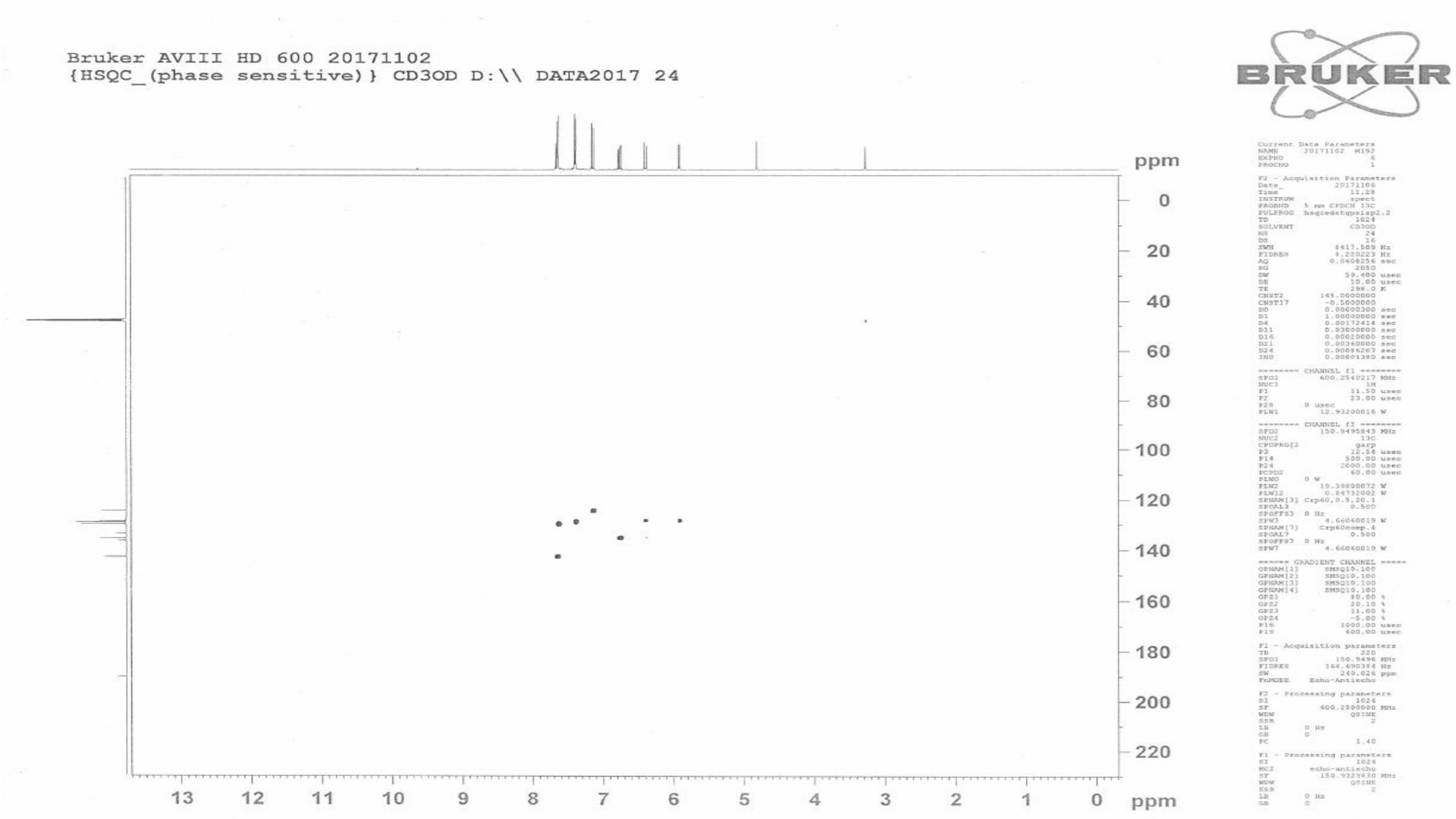

Fig.B.3 HSQC spectrum of synthesized D1

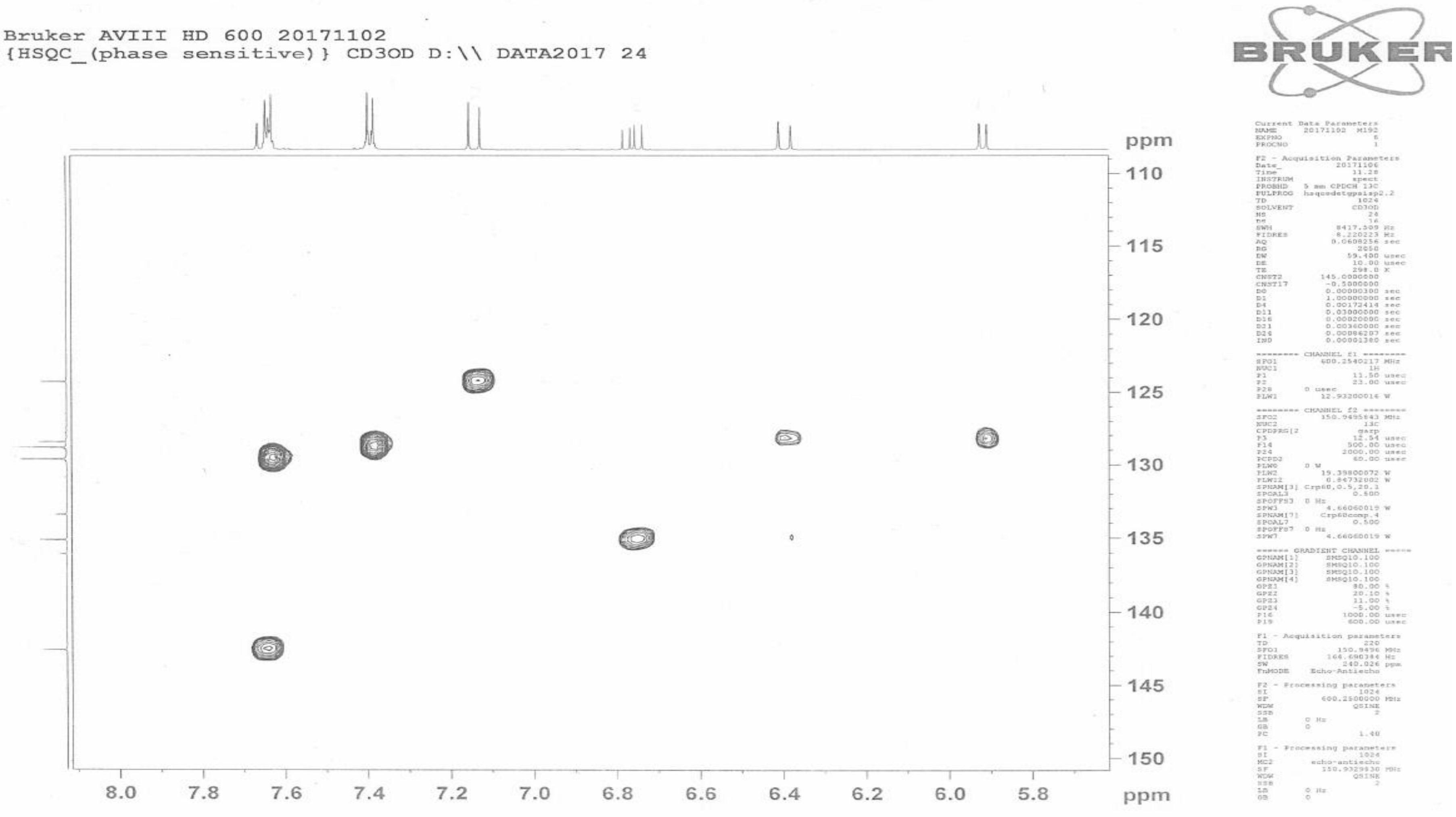

Fig.B.4 partial-enlarged HSQC spectrum of synthesized D1

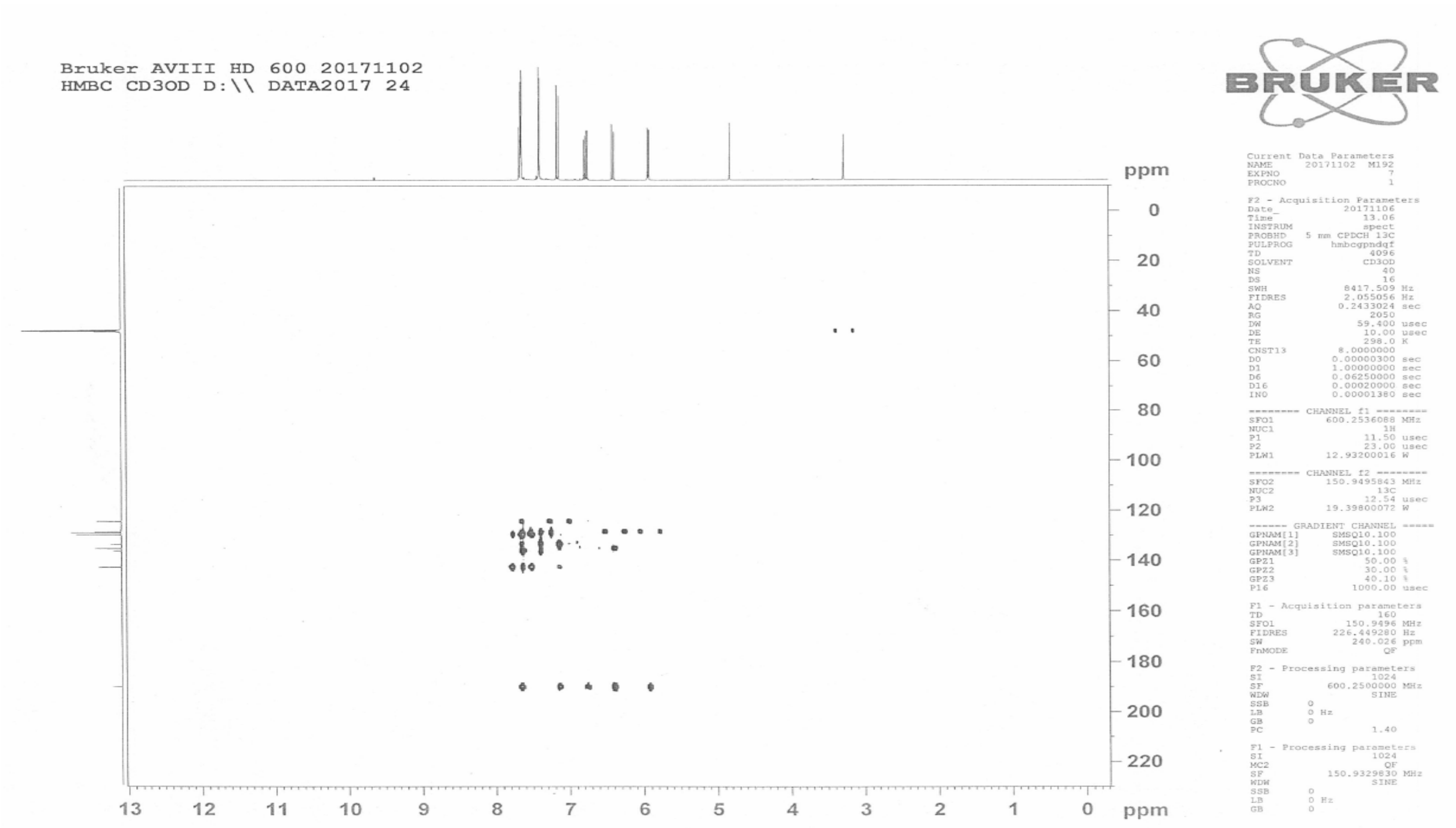

Fig.B.5 HMBC spectrum of synthesized D1

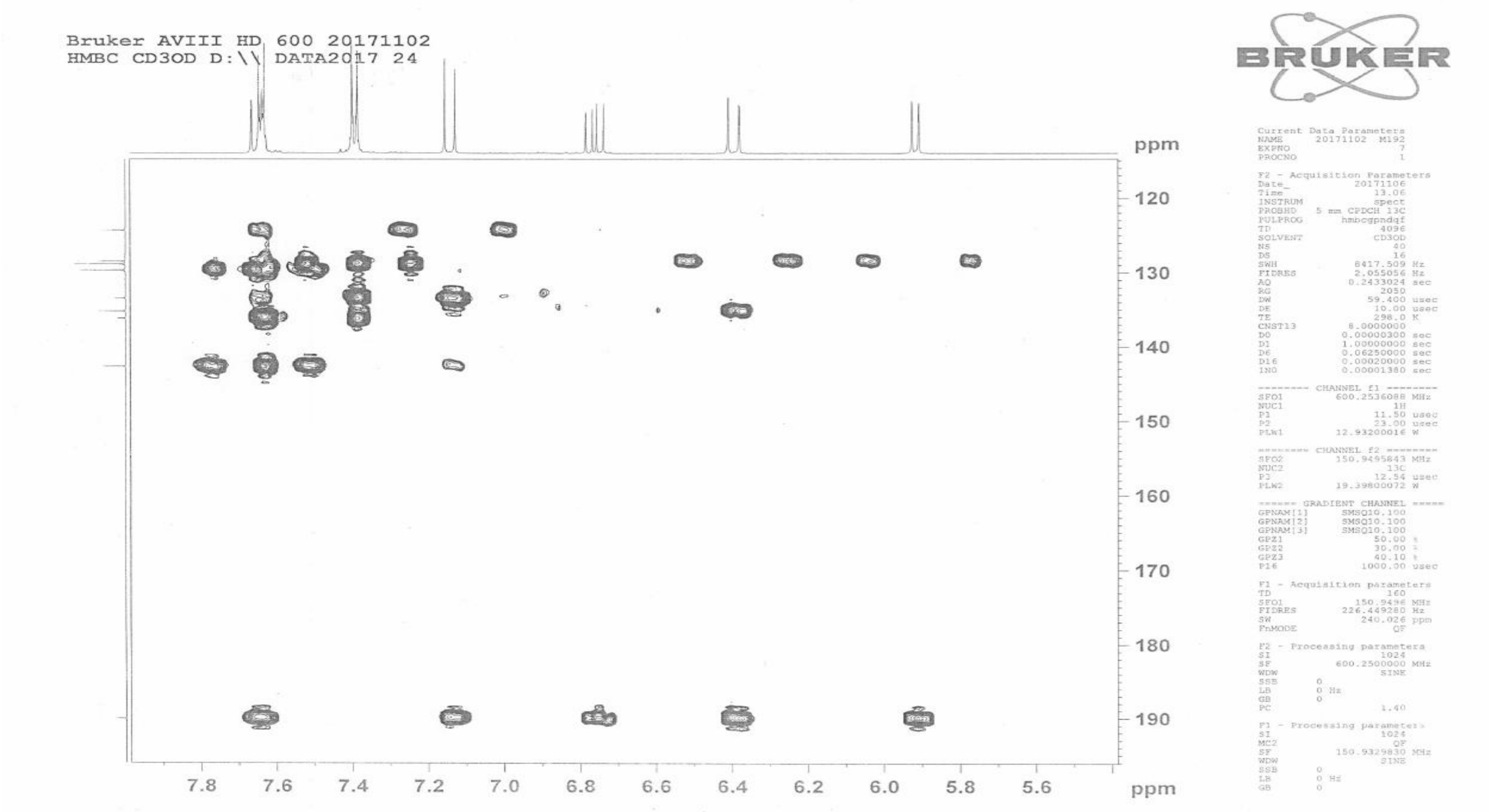

Fig.B.6 partial-enlarged HMBC spectrum of synthesized D1

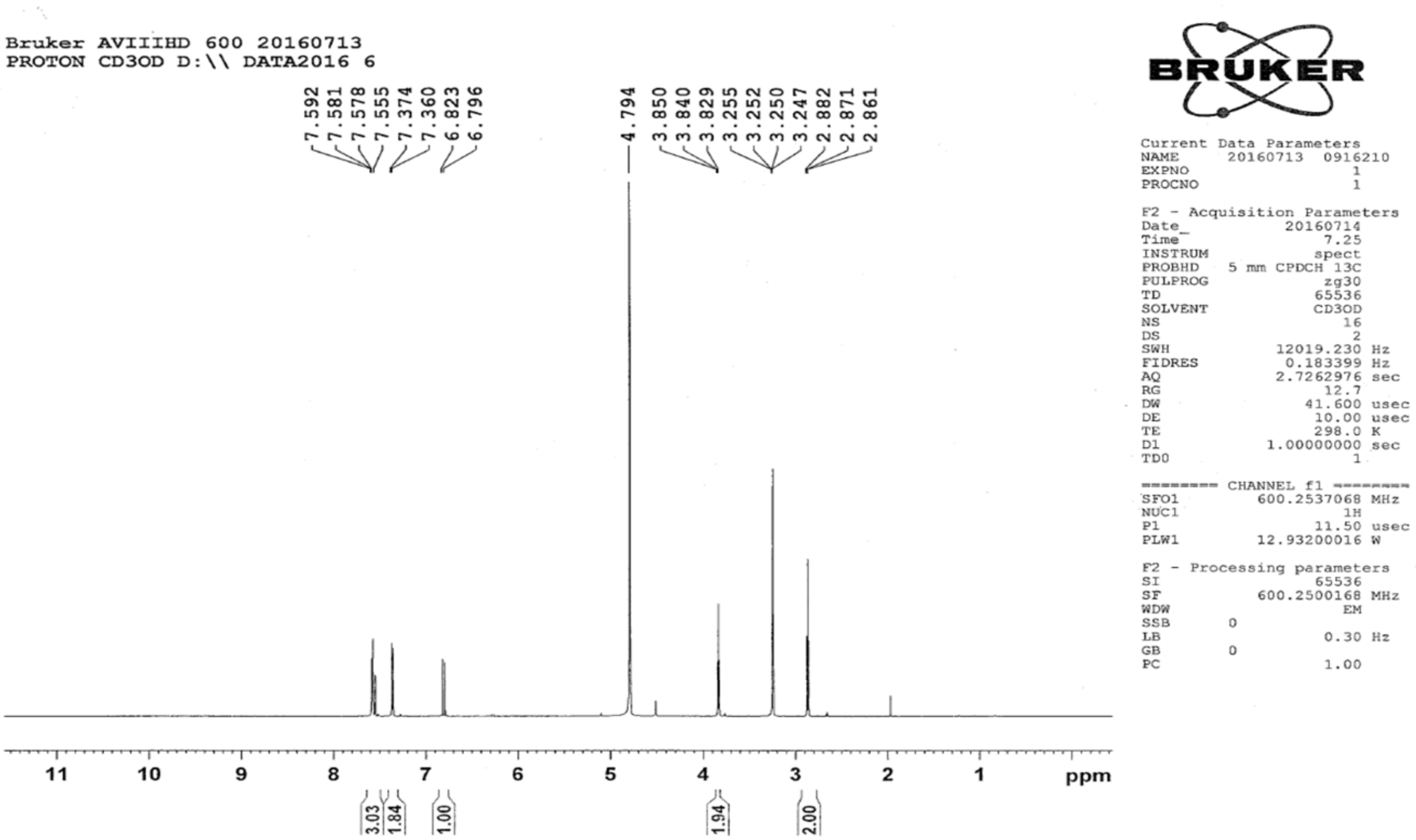

Fig.C.1 ^{1}H spectrum of D2 by LC-SPE-NMR

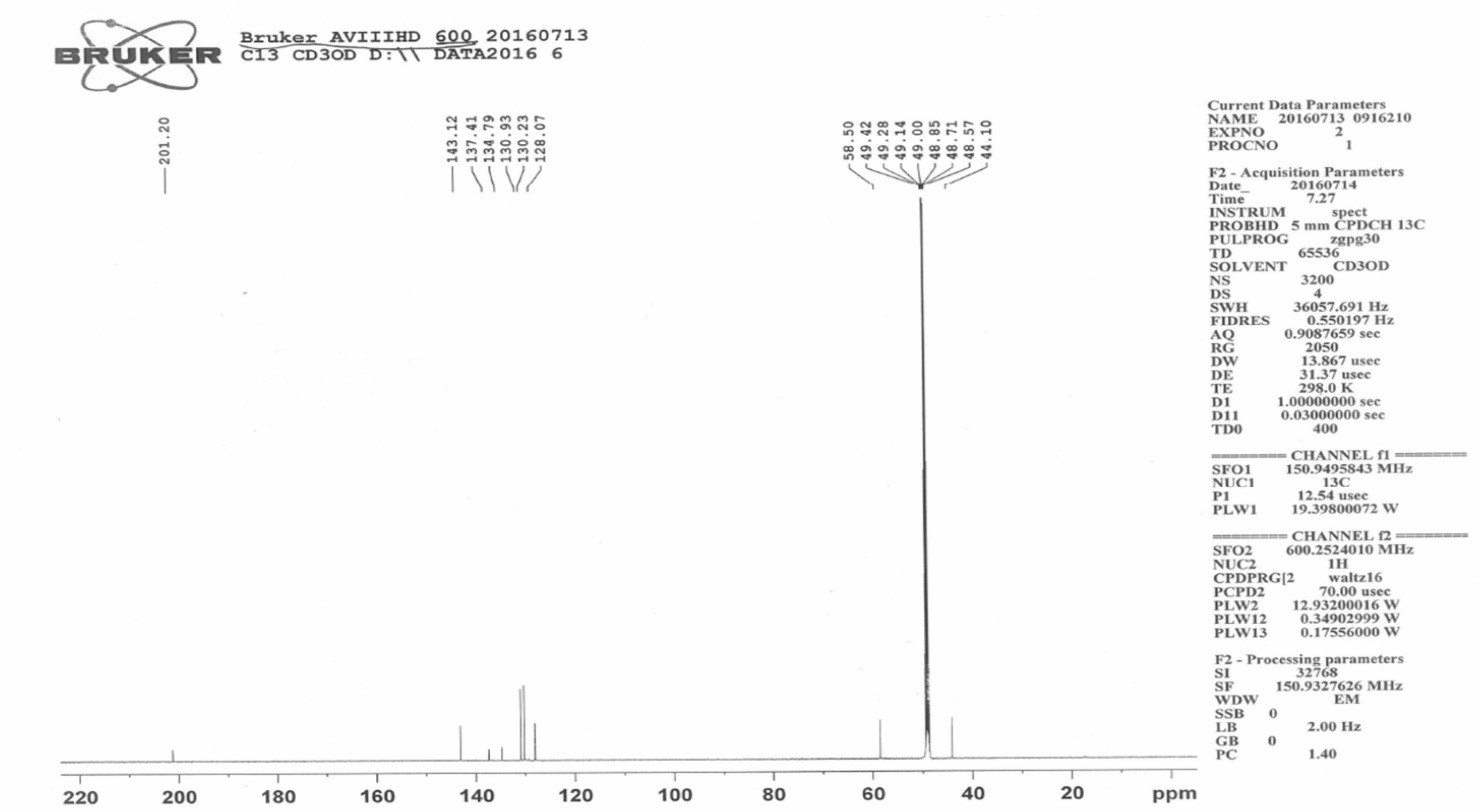

Fig.C.2 ^{13}C spectrum of D2 by LC-SPE-NMR

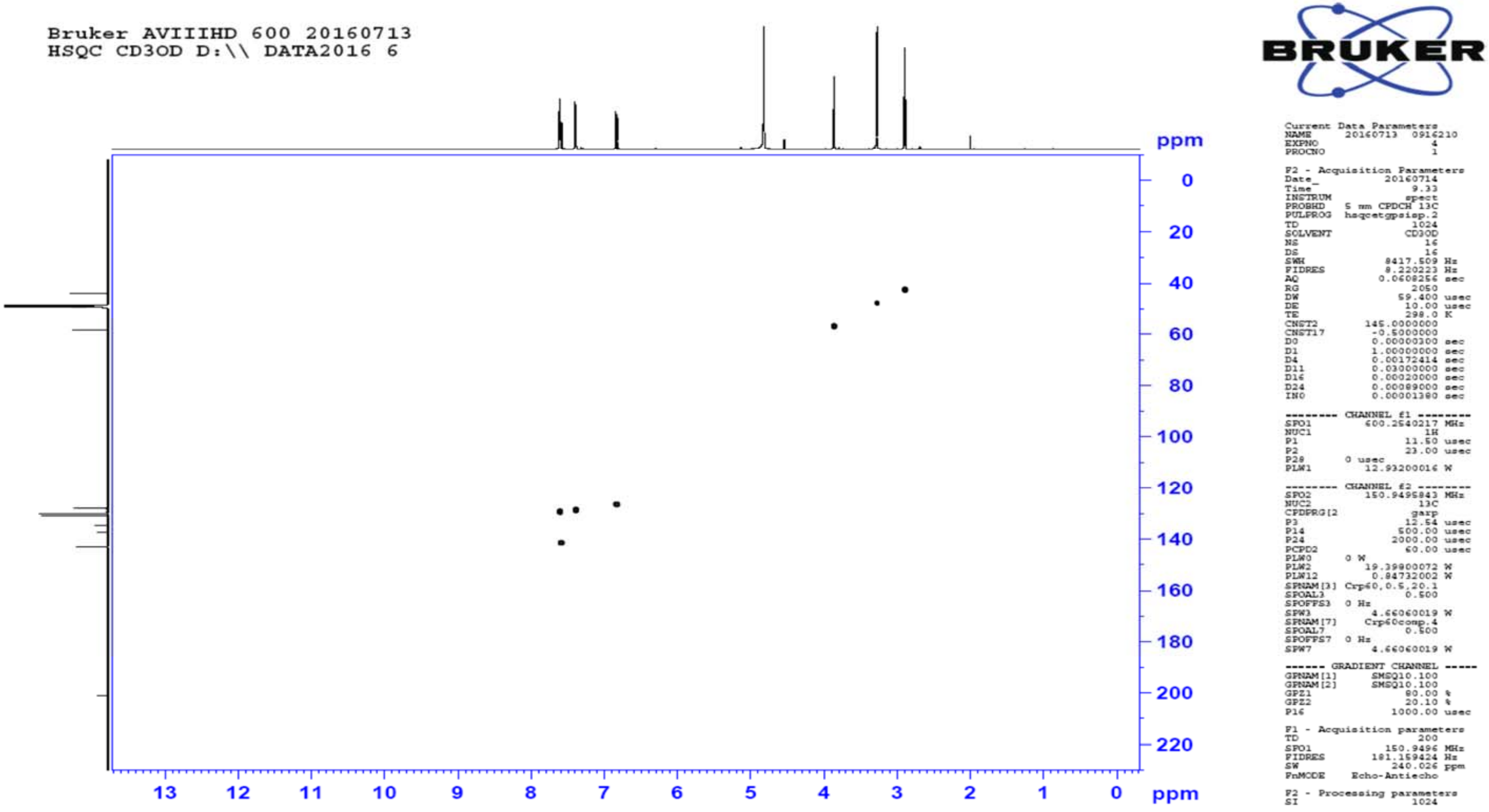

Fig.C.3 HSQC spectrum of D2 by LC-SPE-NMR

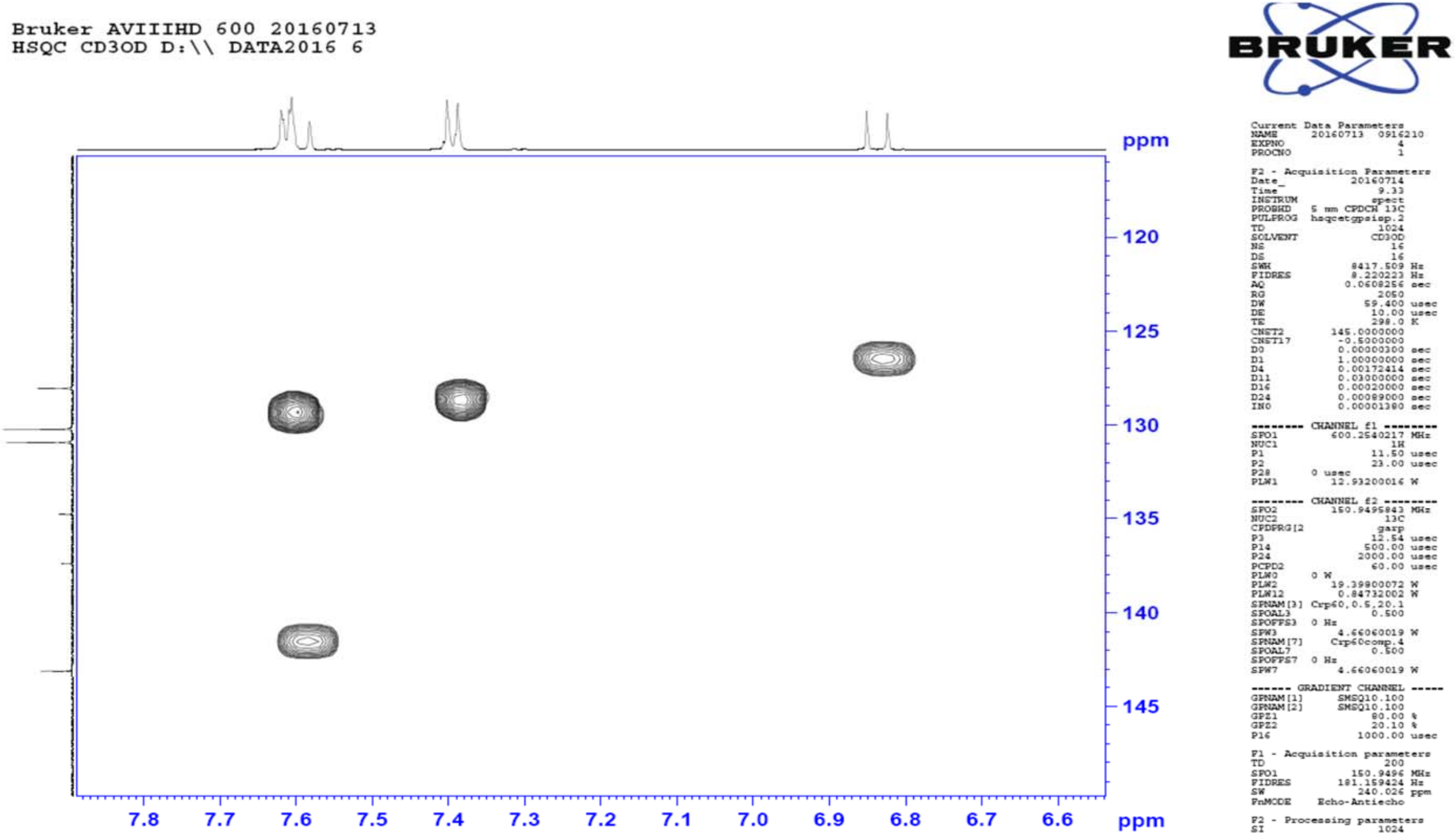

Fig.C.4 partial-enlarged HSQC spectrum of D2 by LC-SPE-NMR

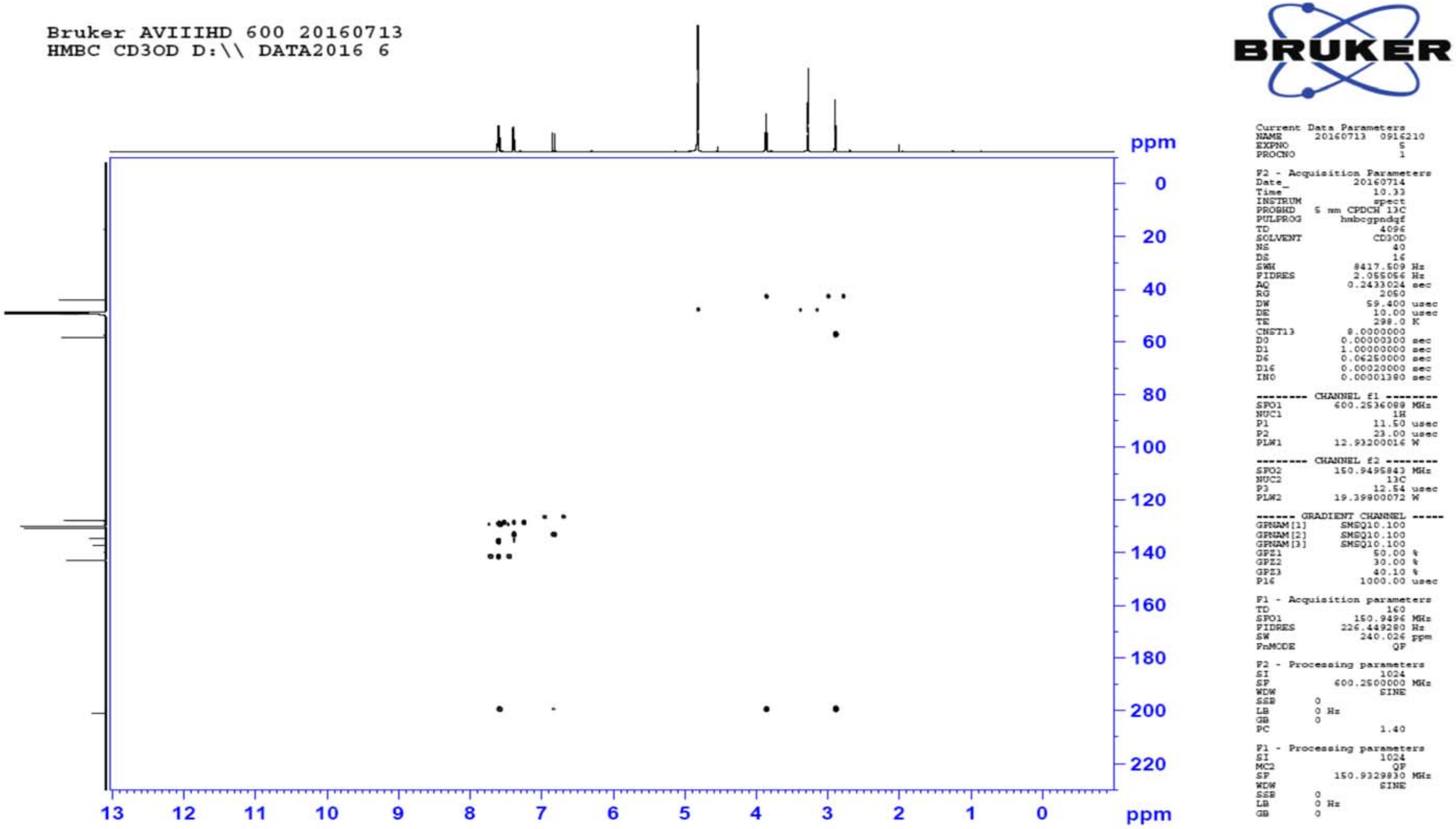

Fig.C.5 HMBC spectrum of D2 by LC-SPE-NMR

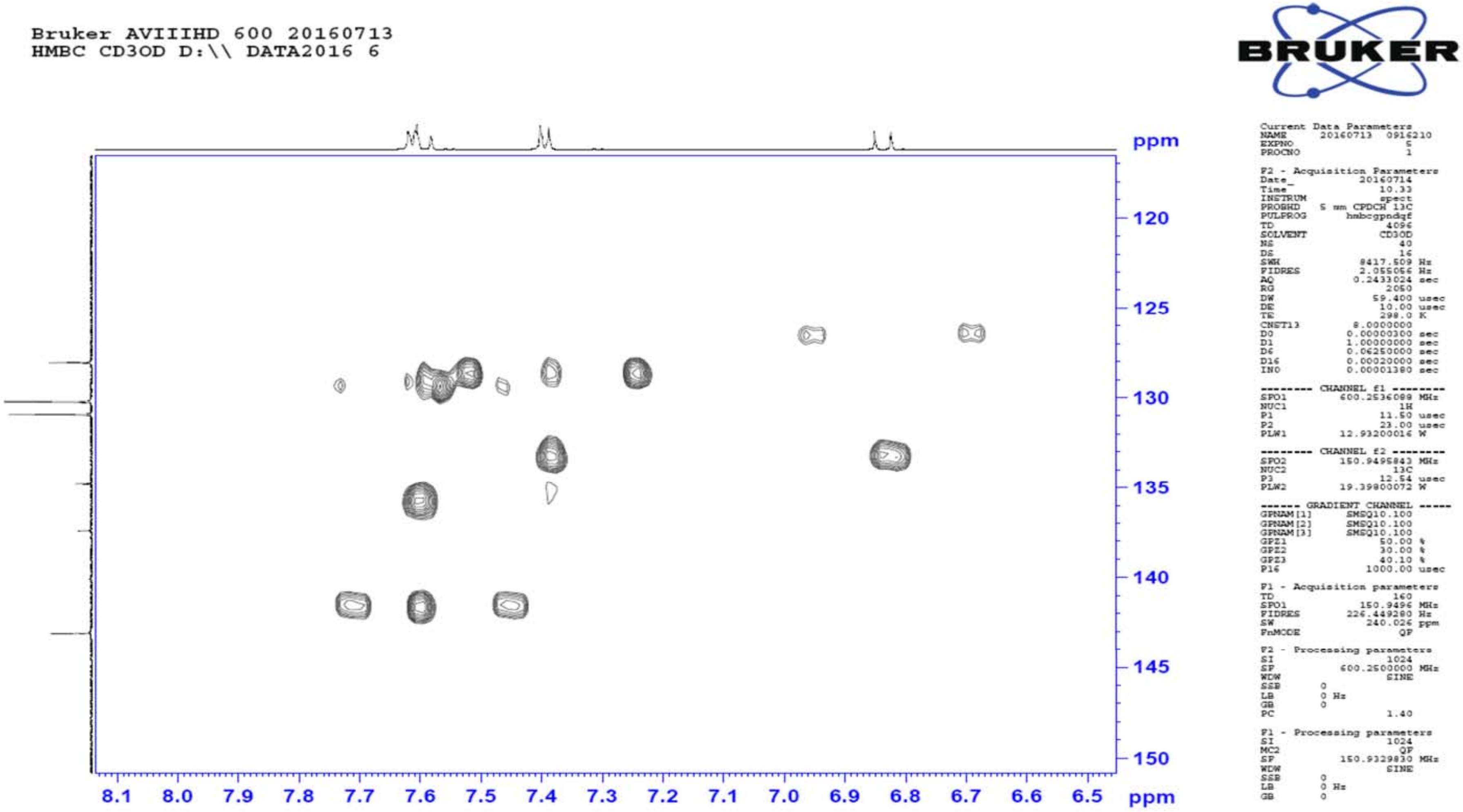

Fig.C.6 partial-enlarged HMBC spectrum of D2 by LC-SPE-NMR
